# Survival Prediction after Curative Resection of Pancreatic Ductal Adenocarcinoma by Imaging-Based Intratumoral Necrosis

**DOI:** 10.3390/cancers14225671

**Published:** 2022-11-18

**Authors:** Hokun Kim, Dong Hwan Kim, In Hye Song, Bohyun Kim, Soon Nam Oh, Joon-Il Choi, Sung Eun Rha

**Affiliations:** 1 Department of Radiology, Seoul St. Mary’s Hospital, College of Medicine, The Catholic University of Korea, 222 Banpo-daero, Seocho-gu, Seoul 06591, Republic of Korea; 2 Department of Pathology, University of Ulsan College of Medicine, Asan Medical Center, 88 Olympic-Ro 43-Gil, Songpa-Gu, Seoul 05505, Republic of Korea

**Keywords:** pancreatic ductal adenocarcinoma, computed tomography, magnetic resonance imaging, necrosis, prognosis

## Abstract

**Simple Summary:**

The histopathological characteristics and prognostic effects of intratumoral necrosis in pancreatic ductal adenocarcinoma (PDAC) observed on computed tomography (CT) or magnetic resonance imaging (MRI) are not well known. This study aimed to determine the histopathological characteristics and prognosis of curatively resected PDAC showing intratumoral necrosis on preoperative CT or MRI. This study consecutively included 102 patients who underwent upfront surgery with margin-negative resection from 2012 to 2020. All patients underwent both pancreatic CT and MRI within 1 month before surgery. PDACs with CT- or MRI-detected necrosis demonstrated a significantly higher degree of histopathological necrosis than those without. Moreover, PDACs with CT- or MRI-detected necrosis were characterized by aggressive histologic tumor grades, higher tumor cellularity, and less frequent remaining acini. Multivariable analysis revealed that only MRI-detected necrosis was significantly associated with overall survival, and therefore MRI-detected necrosis might be a potential imaging predictor of poor survival after curative resection of PDAC.

**Abstract:**

We aimed to determine the histopathological characteristics and prognosis of curatively resected pancreatic ductal adenocarcinoma (PDAC) showing intratumoral necrosis on preoperative CT or MRI. This study consecutively included 102 patients who underwent upfront surgery with margin-negative resection from 2012 to 2020. All patients underwent both pancreatic CT and MRI within 1 month before surgery. Two radiologists independently assessed CT/MRI findings, including the presence of CT- and MRI-detected necrosis. Histopathological characteristics of PDACs according to CT or MRI detection of necrosis were evaluated. Disease-free survival (DFS) and overall survival (OS) were assessed by the Kaplan–Meier method and the Cox proportional hazards model. Among the 102 PDAC patients, 14 patients (13.7%) had CT-detected necrosis, and 16 patients (15.7%) had MRI-detected necrosis, of which 9 showed both CT- and MRI-detected necrosis. PDACs with CT- or MRI-detected necrosis demonstrated a significantly higher degree of histopathological necrosis than those without (*p* < 0.001). Multivariable analysis revealed that tumor size (hazard ratio [HR], 1.19; *p* = 0.040), tumor location (HR, 0.46; *p* = 0.009), and MRI-detected necrosis (HR, 2.64; *p* = 0.002) had independent associations with DFS. Only MRI-detected necrosis was significantly associated with OS (HR, 2.59; *p* = 0.004). Therefore, MRI-detected necrosis might be a potential imaging predictor of poor survival after curative resection of PDAC.

## 1. Introduction

Pancreatic ductal adenocarcinoma (PDAC) is the fourth leading cause of cancer mortality, with an incidence increasing by approximately 1% annually [1]. Due to aggressive biology and late onset of symptoms, the prognosis for PDAC patients is very poor, with a 5-year overall survival (OS) rate of less than 5% [2,3]. Only 20–25% of PDAC patients can be candidates for curative resection, but the 5-year OS rate for patients undergoing margin-negative (R0) resection is only 18–24% [2,4,5]. To improve survival outcomes, practical guidelines currently recommend the implementation of neoadjuvant therapy in patients with high-risk features, even for resectable diseases [6,7]. Thus, it is an important issue to identify preoperative computed tomography (CT) or magnetic resonance imaging (MRI) findings significantly associated with poor clinical outcomes after curative resection.

Histological intratumoral necrosis is an established biomarker for poor prognosis after surgery in PDAC patients [8,9,10]. The presence of necrosis reflects intratumoral hypoxia, and the expression of carbonic anhydrase IX and hypoxia-inducible factor-1α around the necrotic areas are frequently detected by immunohistochemistry [9,10]. In addition to histological necrosis, a few recent studies have shown that intratumoral necrosis on CT or MRI is also an independent poor prognostic factor for resectable PDAC [11,12]. Among several imaging findings that could potentially be used to predict the prognosis of PDAC, imaging necrosis has proven to be one of the significant predictors of disease recurrence after curative resection [11,12]. Thus, the detection of intratumoral necrosis on preoperative CT or MRI is useful for stratifying PDAC patients in terms of the risk of recurrence. However, these studies [11,12] did not fully elucidate the histopathological features of intratumoral necrosis observed on CT (CT-detected necrosis) or MRI (MRI-detected necrosis). In addition, there is no previous study that compared CT-detected necrosis and MRI-detected necrosis in terms of the association with postoperative survival. Therefore, the purpose of this study was to determine the histopathological characteristics and prognosis of curatively resected PDAC showing intratumoral necrosis on preoperative CT or MRI.

## 2. Materials and Methods

This retrospective study was approved by our institutional review board, and informed patient consent was waived due to the study’s retrospective nature.

### 2.1. Study Population

We searched our institution’s electronic database and identified 262 consecutive patients who underwent pancreatic resection with curative intent and had pathological confirmation of PDAC between January 2012 and October 2020. The study population was selected according to the following inclusion criteria: (a) patients without synchronous double primary cancer or a history of extrapancreatic cancer, (b) patients without a history of neoadjuvant treatment prior to surgery, (c) patients with pathologically confirmed R0 (margin-negative resection) PDAC and no evidence of microscopic or gross residual disease (R1 or R2) after surgery, (d) patients who underwent both pancreatic CT and MRI within 1 month before surgery, (e) patients whose pancreatic imaging protocol followed the National Comprehensive Cancer Network (NCCN) guidelines [6], and (f) patients who completed more than 6 months of follow-up after surgery.

### 2.2. Imaging Techniques

Pancreatic CT or MRI scans were performed according to the NCCN guidelines [6]. Pancreatic CT included unenhanced imaging plus pancreatic phase and portal venous phase (PVP) imaging after intravenous contrast injection (Iobrix 300, Taejoon Pharm, Seoul, Korea; Ultravist 300, Bayer Healthcare, Leverkusen, Germany) at a rate of 3–4 mL/s followed by a 20-mL saline flush using an automatic power injector. Pancreatic phase images were obtained at 12 s after the abdominal aorta reached 100 Hounsfield units using a bolus-triggering technique, followed by PVP at a delay of 40 s. CT images were obtained with a 64- or 128-slice CT scanner (Somatom Sensation 64/Definition/Definition AS+, Siemens Healthineers, Erlangen, Germany; Discovery CT750 HD, GE Healthcare, Milwaukee, WI, USA) using a slice thickness of 2–3 mm with no gap. MR images were obtained with a 3.0-T scanner (Magnetom Verio/Vida, Siemens Healthineers, Erlangen, Germany; Ingenia CX, Philips Healthcare, Best, Netherlands). Pancreas MRI consisted of dual-echo in- and out-of-phase imaging, breath-hold single-shot fast spin-echo T2-weighted imaging (T2WI), navigator-triggered fat-suppressed fast spin-echo T2WI, diffusion-weighted imaging using two *b* values (0 and 800 s/mm^2^), and T1-weighted imaging before and after contrast agent injection. For dynamic contrast-enhanced imaging, three-dimensional gradient-recalled echo imaging was performed before contrast injection and during the pancreatic phase (30−35 s after contrast injection using the bolus-triggering technique), PVP (65−80 s), and delayed phases (3 min and 5 min). Gadoterate meglumine (0.2 mL/kg; Dotarem, Guerbet, Aulnay-sous-Bois, France) was used as a contrast agent at an injection rate of 2.0 mL/s followed by a 20-mL saline flush using a power injector. Details of the MRI parameters are described in Supplementary Table S1.

### 2.3. Imaging Analysis

Two board-certified abdominal radiologists (with more than 6 years of experience in pancreatic imaging) independently reviewed the preoperative CT or MR images. Both reviewers were aware that all patients had PDAC, but they were blinded to the clinical data, histopathological results, and postoperative outcomes. Disagreements between the two reviewers were resolved by reanalysis and, if necessary, discussion with another senior radiologist (with 24 years of experience in pancreatic imaging) to reach a consensus.

**Figure 1:**
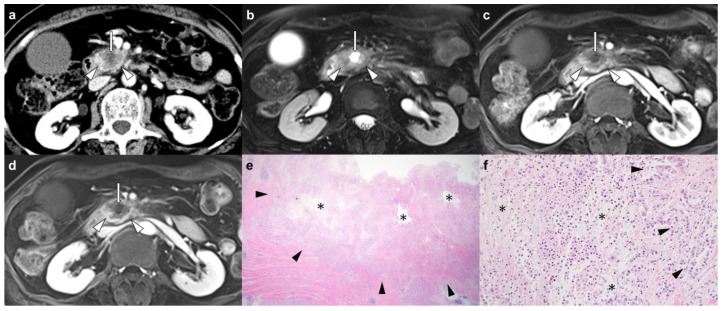
Pancreatic ductal adenocarcinoma in the head of the pancreas in a 65-year-old woman. (**a**) An axial contrast-enhanced CT image shows a 4.5 cm hypoenhancing pancreatic head mass (arrowheads) with centrally located nonenhanced tumoral tissue judged to be the presence of CT-detected necrosis (arrow). (**b**) An axial T2-weighted MR image with fat suppression shows a pancreatic head mass (arrowheads) accompanied by an intratumoral fluid-containing area with a bright signal intensity at the central location (arrow). On axial dynamic contrast-enhanced T1-weighted MR images, (**c**) portal-venous and (**d**) delayed phases show a hypoenhancing pancreatic head mass (arrowheads) with nonenhanced intratumoral tissue, which is judged to be MRI-detected necrosis (arrow). (**e**,**f**) Micrographs show intratumoral necrosis (asterisks) surrounded by carcinoma cells forming glands (black arrowheads) (hematoxylin and eosin stain, ×12.5 (**e**) and ×200 (**f**)). In this patient, the presence of imaging necrosis was consistent between CT and MRI, and areas of necrosis accounted for approximately 20% of tumors based on histopathology. Local tumor recurrence was detected on follow-up CT 2 months after margin-negative resection (not shown), and the patient died 6 months later.

The reviewers assessed the presence of necrosis within PDAC on preoperative CT or MR images. CT-detected necrosis was defined as intratumoral tissue that did not reach 15 Hounsfield unit threshold enhancement on pancreatic phase or PVP images compared to unenhanced images (Figure 1) [11,13]. MRI-detected necrosis was defined as an intratumoral fluid-containing area showing a cerebrospinal fluid-like high signal intensity on T2WI, and no contrast enhancement on dynamic-enhanced images (Figure 1 and Supplementary Figure S1) [12,13,14,15]. Both CT- and MRI-detected necrosis are centrally located within the PDAC and have irregular margins (Figure 1 and Supplementary Figure S1) [12,13,14,15]. Other imaging features to be analyzed were selected based on their reported likelihood as predictors of postoperative prognosis in PDAC patients [11,16,17]. First, tumor contact with the portal vein or superior mesenteric vein was assessed on CT or MRI. Second, tumor signal intensity was compared with the unaffected pancreatic parenchyma on the T1-weighted unenhanced, dynamic-enhanced pancreatic phase, PVP, and delayed phase MR images (hypointensity versus hyper or isointensity). Third, the presence of rim enhancement was assessed, defined as irregular rim-like enhancement with a relatively hypoenhancing central area on dynamic-enhanced MRI [17]. Fourth, the presence of diffusion restriction, defined as high signal intensity on DWI (*b* = 800 s/mm^2^) and low signal intensity on an apparent diffusion coefficient (ADC) map of the corresponding tumor, was evaluated. Fifth, the ADC value at the level of the largest cross-sectional area of the tumor was quantitatively measured. Each reviewer measured and averaged the ADC values in two manually drawn regions of interest within the tumor after excluding necrosis. The mean ADC value of the two reviewers was used for the analysis of this study.

### 2.4. Clinical, Histopathological, and Follow-Up Data Collection

Clinical and histopathological variables were collected from patients’ electronic medical records by one of the authors who was not involved in the image analysis. Clinical variables included age, sex, and serum concentrations of carbohydrate antigen (CA) 19-9 measured within 1 month before surgery. Histopathological variables included tumor location (head/uncinate process, body, or tail of the pancreas), tumor size, tumor stage of PDAC according to the 8th edition of the American Joint Committee on Cancer (AJCC) staging system [18], tumor differentiation, and the presence of lymphovascular or perineural invasion. In addition, a pathologist (with 9 years of experience in pathological examination of pancreatic disease) who was blinded to the clinical and radiological findings retrospectively reviewed the histopathological slides of all patients to evaluate the following pathological findings: (a) the quantitative degree of histopathological necrosis within the tumor (%); (b) tumor cellularity (%); and (c) the presence of remaining acini [19].

Postoperative contrast-enhanced pancreatic CT or MRI and serum CA 19-9 levels were routinely checked every 3–6 months after surgery for surveillance of recurrence [6]. Disease-free survival (DFS) was defined as the time from the date of surgery to the date of recurrence on follow-up CT or MRI or death [20]. Overall survival (OS) was defined as the time from the date of surgery to the date of death.

### 2.5. Statistical Analysis

Categorical variables are shown as numbers with frequencies and were compared using χ^2^ or Fisher’s exact tests. Continuous variables were summarized as the means with standard deviations and compared using Mann–Whitney U or Student’s *t* tests, depending on normal distribution (tested by Kolmogorov–Smirnov test). The diagnostic accuracy of CT- or MRI-detected necrosis was evaluated using histopathological necrosis as a reference standard. DFS and OS rates for subgroups (PDAC patients with or without CT-detected necrosis and PDAC patients with or without MRI-detected necrosis) were estimated using the Kaplan–Meier method and compared using log-rank tests. In addition, to identify independent variables significantly predictive of postoperative recurrence or death, univariable and multivariable Cox proportional hazards models were used. Variables with a *p* value < 0.050 in univariable analysis were included in the multivariable analysis. Interreader agreement between the two reviewers in imaging analysis was assessed using the overall proportion of agreement, free-marginal κ statistics, and intraclass correlation coefficient (ICC). Statistical analyses were performed using R version 3.6.3 (The R Foundation for Statistical Computing), with *p* < 0.050 indicating a significant difference.

## 3. Results

### 3.1. Summary of Patient Characteristics

**Figure 2:**
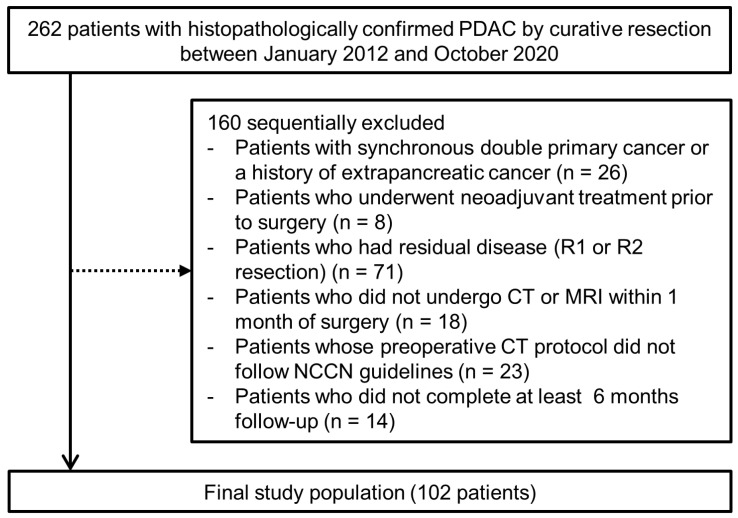
Diagram of the study population.

After applying eligibility criteria, the final study population consisted of 102 patients, and details of the flow diagram are presented in Figure 2. Among the 102 patients, imaging findings of 83 patients have been reported previously [12]. However, the previous study focused on the prognostic implication of intratumoral fluid-containing areas observed on MRI. In contrast, our study aimed to evaluate the postoperative outcomes of PDACs showing intratumoral necrosis on CT or MRI with histopathologic correlation. Of a total of 102 PDAC patients (mean age, 64.9 ± 10.2 years; 51 men and 51 women), 14 (13.7%) patients had CT-detected necrosis, and 16 (15.7%) patients had MRI-detected necrosis, of which 9 showed both CT- and MRI-detected necrosis. Further details on the consistency between CT- and MRI-detected necrosis are described in Supplementary Material. The mean diameter of PDACs was 3.3 ± 1.2 cm, and the most common location of PDACs was the head/uncinate process of the pancreas (76.5%, 78/102). According to the 8th edition of the AJCC classification, 37 (36.3%) patients had stage I, 50 (49.0%) had stage II, and 15 (14.7%) had stage III disease. The clinicopathological and radiological data of all patients and their comparisons according to CT- or MRI-detected necrosis are provided in Table 1. A larger tumor size was observed in both patients with CT-detected necrosis (4.3 versus 3.1 cm, *p* = 0.001) and MRI-detected necrosis (3.9 versus 3.2 cm, *p* = 0.031) than in those without. Among the radiological findings, tumor contact with SMV or PV (71.4 versus 38.6%, *p* = 0.021), delayed phase hypointensity (100 versus 63.6%, *p* = 0.004), and rim enhancement on MRI (92.9 versus 31.8%, *p* < 0.001) were more frequently observed in patients with CT-detected necrosis than in those without it. A higher frequency of delayed phase hypointensity (100 versus 62.8%, *p* = 0.003) and rim enhancement on MRI (92.9 versus 29.1%, *p* < 0.001) was also found in patients with MRI-detected necrosis than in those without it.

### 3.2. Comparison of Histopathological Findings According to Imaging Necrosis

Table 2 summarizes the histopathological characteristics according to imaging necrosis. PDACs with CT- or MRI-detected necrosis were characterized by more frequent histopathologic tumor necrosis than those without (CT-detected necrosis, *p* = 0.002; MRI-detected necrosis, *p* < 0.001). In addition, the quantitative degree of histopathological necrosis was significantly higher in PDACs with CT- or MRI-detected necrosis than in those without (18.6 versus 5.9%, *p* < 0.001 for CT-detected necrosis; 20.0 versus 5.3%, *p* < 0.001 for MRI-detected necrosis). When histopathologic necrosis served as the reference standard, the sensitivity, specificity, and odds ratio of CT-detected necrosis were 24.5%, 96.2%, and 8.3 (95% CI [confidence interval], 1.75–39.19), respectively, and those of MRI-detected necrosis were 30.6%, 98.1%, and 22.9 (95% CI, 2.9–181.78), respectively. PDACs with CT- or MRI-detected necrosis were significantly associated with aggressive histologic tumor grades, higher tumor cellularity, and less frequent remaining acini than those without (*p* < 0.050). Supplementary Table S2 shows the comparison of histopathological characteristics of CT-detected necrosis according to contrast agent.

### 3.3. Postoperative Outcomes According to Imaging Necrosis

Of the 102 patients followed up, we were unable to determine whether 2 were alive or not. Tumor recurrence and death were identified in 80 (78.4%) and 60 (60.0%) patients during the follow-up period (median, 23 months; range, 6–82 months), respectively. Local tumor recurrence (*n* = 33) was the most common recurrence, followed by liver metastasis (*n* = 20), peritoneal seeding (*n* = 12), lung metastasis (*n* = 8), and lymph node metastasis (*n* = 7). The cumulative 1-, 3-, and 5-year DFS rates were 81.4%, 62.7%, and 42.5%, respectively. The cumulative 1-, 3-, and 5-year OS rates were 82.0%, 69.1%, and 54.2%, respectively.

**Figure 3:**
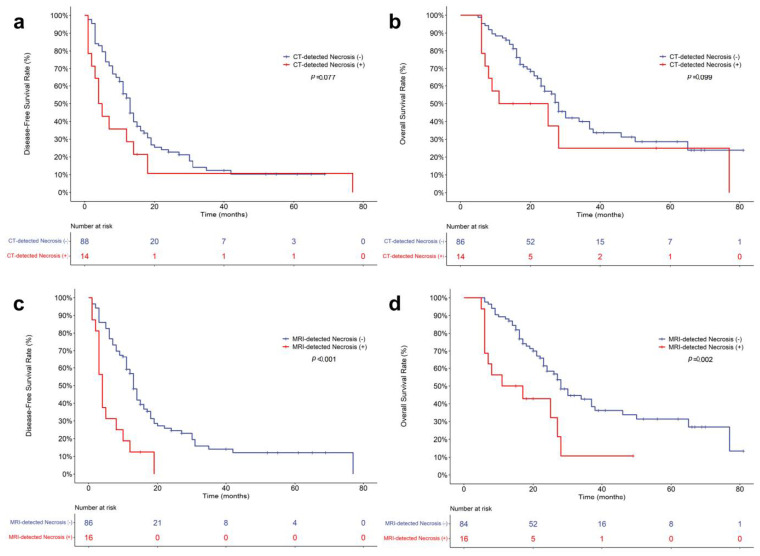
Kaplan–Meier curves for postoperative survival outcomes of pancreatic ductal adenocarcinoma patients. Comparison of disease-free survival (**a**) and overall survival (**b**) according to the presence of CT-detected necrosis. Comparison of disease-free survival (**c**) and overall survival (**d**) according to the presence of MRI-detected necrosis.

In a log-rank analysis based on Kaplan–Meier curves, the DFS and OS rates were significantly worse in patients with MRI-detected necrosis than in those without it (DFS, *p* < 0.001; OS, *p* = 0.002) (Figure 3). The mean DFS and OS of PDAC patients with MRI-detected necrosis were 6.3 and 18.5 months, respectively, and those of PDAC patients without MRI-detected necrosis were 21.3 and 40.0 months, respectively. The DFS and OS rates tended to be poorer in patients with CT-detected necrosis than those without it, but there were no significant differences (DFS, *p* = 0.077; OS, *p* = 0.099) (Figure 3). The mean DFS and OS of PDAC patients with CT-detected necrosis were 14.0 and 29.7 months, respectively, and those of PDAC patients without CT-detected necrosis were 19.1 and 38.7 months, respectively. Survival outcomes in the subgroups according to CT contrast agent are described in Supplementary Material.

### 3.4. Univariable and Multivariable Analyses to Identify Predictors of Postoperative Outcomes

Table 3 and Table 4 demonstrate the association of clinical, pathological, and imaging findings with survival using univariable and multivariable Cox regression analyses. MRI-detected necrosis had a higher HR than CT-detected necrosis for DFS (3.16 [95% CI, 1.77–5.65] versus 1.71 [0.93–3.16]) and OS (2.66 [1.40–5.06] versus 1.79 [0.90–3.56]) in univariable analysis, and CT-detected necrosis was not a significant predictor of worse survival. Univariable analysis revealed tumor size, tumor location, LN metastasis, MRI-detected necrosis, delayed phase hypointensity, and diffusion restriction to be significantly associated with DFS (*p* < 0.050). OS showed similar results, but tumor location, delayed phase hypointensity, and diffusion restriction were not statistically significant in univariable analysis. On multivariable analysis, tumor size (per cm; HR, 1.19 [95% CI, 1.01–1.41], *p* = 0.040), tumor location (body and tail; HR, 0.46 [95% CI.25–.82], *p* = 0.009), and MRI-detected necrosis (HR, 2.64 [95% CI, 1.43–4.89], *p* = 0.002) showed significant independent associations with DFS, and only MRI-detected necrosis (HR, 2.59 [95% CI, 1.35–4.97], *p* = 0.004) was an independent factor for predicting worse OS.

### 3.5. Interreader Agreement for Imaging Analysis

The interreader agreement for the presence or absence of CT- or MRI-detected necrosis was substantial (κ, 0.76−0.80), with the overall proportion of reader agreement ranging from 88.2% to 90.2%. Regarding other qualitative imaging analyses, there was a moderate to a substantial agreement (κ, 0.49−0.76; the overall proportion of agreement, 74.5−88.2%) with the exception of the fair agreement for rim enhancement on MRI (κ, 0.37; the overall proportion of agreement, 68.6%). The quantitative measurement of the ADC showed excellent reliability (ICC, 0.89). Further details on the interreader agreement are presented in Supplementary Table S3.

## 4. Discussion

Our study demonstrated that PDAC with CT- or MRI-detected necrosis was significantly correlated with histopathological necrosis and was also characterized by other aggressive histologic features. Moreover, imaging necrosis appears to be highly reproducible, given that the assessment of imaging necrosis was achieved with substantial interreader agreement (κ, 0.76−0.80). Although both CT- and MRI-detected necrosis were associated with poor survival rates after curative resection of PDAC, multivariable analysis revealed that only MRI-detected necrosis had a significant independent association with DFS (HR, 2.64; *p* = 0.002) and OS (HR, 2.59; *p* = 0.004). These results indicate that imaging necrosis found on preoperative MRI can be used to predict the postoperative prognosis of PDAC patients.

Our data showed that imaging necrosis of PDAC detected on preoperative CT or MRI qualitatively and quantitatively correlated with histopathologic necrosis. The significant association between imaging and histopathologic necrosis may explain the more aggressive tumor differentiation, higher cellularity, and less frequent remaining acini of PDACs with imaging necrosis compared to those without imaging necrosis. These results aligned well with prior studies that attempted radio-pathological correlations of necrosis in PDAC [12,13,14]. Although both CT- and MRI-detected necrosis in PDAC had a strong association with histopathologic tumor necrosis, the diagnostic performance of MRI-detected necrosis for predicting histopathologic necrosis was higher than that of CT-detected necrosis (odds ratio, 22.9 versus 8.3). This result might be attributable to the fact that MRI can provide excellent soft-tissue contrast, especially for fluid-containing areas using heavily T2WI. However, to directly compare the histopathologic characteristics of CT- and MRI-detected necrosis, further studies using a larger number of PDACs showing imaging necrosis are warranted.

The DFS and OS of PDAC patients with imaging necrosis were longer than those without imaging necrosis, but only MRI-detected necrosis showed statistical significance. It is worth emphasizing that MRI-detected necrosis was a significant prognostic factor associated with poor postoperative survival according to our multivariable analysis. Several previous studies have reported that imaging necrosis on CT or MRI was a poor independent predictor of disease recurrence in patients with PDAC regardless of the presence of histopathologic necrosis [11,12]. However, our study is the first to demonstrate that MRI-detected necrosis is a superior prognostic factor to CT-detected necrosis and is an independent factor significantly related to OS and DFS. In addition to the fact that MRI has comparable performance to CT in determining the resectability of PDAC [21,22], our results support the need for pancreatic MRI as a preoperative workup to detect intratumoral necrosis. If a PDAC patient with MRI-detected necrosis undergoes pancreatic resection, the patient may be alerted to an increased risk of disease recurrence after surgery, and shorter follow-up intervals may be considered for postoperative surveillance using CT or MRI and serum CA 19-9 levels. According to recently updated NCCN guidelines [6], neoadjuvant therapy is recommended for selective patients who appear technically resectable but have poor prognostic factors, including imaging features. Considering that there is currently limited evidence for imaging features that increase the risk of poor postoperative outcomes, our results may be worthwhile. Future clinical trials are necessary to determine whether neoadjuvant therapy for PDAC patients with MRI-detected necrosis truly leads to benefits in terms of postoperative survival and cost-effectiveness.

Multivariable analysis of our study showed that, in addition to MRI-detected necrosis, tumor size and tumor location were significantly associated with DFS. Many previous studies investigating the prognostic factors of PDAC have already revealed that tumor size is an important factor in predicting clinical outcomes [23]. Our findings are also in accordance with previous studies that reported higher survival rates in pancreatic body/tail cancer patients than in pancreatic head cancer patients at a resectable early stage [24,25,26].

In our study, there were several limitations. First, the study had a retrospective design, which may be subject to selection bias. Second, this study only included patients who underwent margin-negative (R0) resection for PDAC. Although this inclusion criterion may be associated with a selection bias by excluding a substantial number of patients (*n* = 71) with residual disease after surgery (R1 or R2 resection), it enables a more reliable prognostic analysis by controlling resection margin status, which is a well-known confounding factor for survival rates of PDAC patients [27,28]. To mitigate the effects of selection bias, we consecutively enrolled patients who met the eligibility criteria. Future studies involving R1 or R2 resection groups are needed to develop a preoperative prediction model using imaging and clinical findings. Third, the statistical power may be limited by a small number of PDAC patients, especially those with imaging necrosis. Fourth, our study population was enrolled from a single institution, and validation using datasets from other institutions was not performed. A multicenter prospective study with a larger study population is needed to confirm our findings.

## 5. Conclusions

Overall, intratumoral necrosis in PDAC found on CT or MRI was significantly correlated with histopathological necrosis as well as aggressive tumor differentiation, higher cellularity, and less frequent remaining acini. With respect to recurrence or survival after R0 resection of PDAC, only MRI-detected necrosis was identified as a poor independent predictor. Therefore, MRI-detected necrosis may be clinically useful for predicting poor outcomes after curative resection of PDAC and is feasible as a biomarker for risk stratification in terms of the postoperative prognosis of patients with resectable PDAC.

## Figures and Tables

**Table 1:** Summary of clinical, pathological, and radiological findings of PDAC patients.

Variable	Total (*n* = 102)	PDAC with CT-Detected Necrosis			PDAC with MRI-Detected Necrosis		
		Yes (*n* = 14)	No (*n* = 88)	*p*	Yes (*n* = 16)	No (*n* = 86)	*p*
Age, years *	64.9 ± 10.2	61.5 ± 10.9	65.4 ± 10.1	0.183	62.8 ± 12.6	65.3 ± 9.7	0.378
Sex (male:female)	51:51	8:6	43:45	0.774	9:7	42:44	0.785
Serum CA 19-9, U/mL *	638 ± 1284	903 ± 1615	594 ± 1226	0.408	1125 ± 2219	543 ± 1003	0.319
Tumor size, cm *	3.3 ± 1.2	4.3 ± 0.9	3.1 ± 1.2	0.001	3.9 ± 1.1	3.2 ± 1.2	0.031
Location				0.887			0.524
Head/uncinate process	78 (76.5)	11 (78.6)	67 (76.1)		14 (87.4)	64 (74.4)	
Body	11 (10.9)	1 (7.1)	10 (11.4)		1 (6.3)	10 (11.6)	
Tail	13 (12.7)	2 (14.3)	11 (12.5)		1 (6.3)	12 (14.0)	
T stage				0.538			0.382
T1	27 (26.5)	2 (14.3)	25 (28.4)		2 (12.5)	25 (29.1)	
T2	69 (67.6)	11 (78.6)	58 (65.9)		13 (81.2)	56 (65.1)	
T3	6 (5.9)	1 (7.1)	5 (5.7)		1 (6.3)	5 (5.8)	
N stage				0.784			0.213
N0	37 (36.3)	4 (28.6)	33 (37.5)		4 (25.0)	33 (38.4)	
N1	50 (49.0)	8 (57.1)	42 (47.7)		11 (68.7)	39 (45.3)	
N2	15 (14.7)	2 (14.3)	13 (14.8)		1 (6.3)	14 (16.3)	
Contact with SMV or PV	44 (43.1)	10 (71.4)	34 (38.6)	0.021	10 (62.5)	34 (39.5)	0.089
Unenhanced T1WI hypointensity	95 (93.1)	14 (100.0)	81 (92.0)	0.589	16 (100.0)	79 (91.9)	0.593
Pancreatic phase hypointensity	94 (92.2)	14 (100.0)	80 (90.9)	0.522	16 (100.0)	78 (90.7)	0.445
Portal venous phase hypointensity	85 (83.3)	14 (100.0)	71 (80.7)	0.157	16 (100.0)	69 (80.2)	0.113
Delayed phase (3 min) hypointensity	70 (68.6)	14 (100.0)	56 (63.6)	0.004	16 (100.0)	54 (62.8)	0.003
Diffusion restriction †				0.295			0.724
Absence	12 (11.8)	0 (0.0)	12 (14.0)		1 (6.3)	11 (13.1)	
Presence	88 (88.2)	14 (100.0)	74 (86.0)		15 (93.7)	73 (86.9)	
ADC (×10^−3^ mm^2^/s) *	1.37 ± 0.38	1.19 ± 0.21	1.40 ± 0.39	0.062	1.28 ± 0.51	1.38 ± 0.34	0.323
Rim enhancement on MRI	41 (40.2)	13 (92.9)	28 (31.8)	<0.001	16 (100.0)	25 (29.1)	<0.001

Values are presented as the number (%) of patients unless indicated otherwise. ADC, apparent diffusion coefficient; CA, carbohydrate antigen; CT, computed tomography; MRI, magnetic resonance imaging; PDAC, pancreatic ductal adenocarcinoma; PV, portal vein; SMV, superior mesenteric vein; T1WI, T1-weighted image. * Values are the mean ± standard deviation. † Diffusion-weighted imaging was unavailable for two patients.

**Table 2:** Summary of histopathologic findings of PDAC according to imaging necrosis.

Variable	Total (*n* = 102)	PDAC with CT-Detected Necrosis			PDAC with MRI-Detected Necrosis		
		Yes (*n* = 14)	No (*n* = 88)	*p*	Yes (*n* = 16)	No (*n* = 86)	*p*
Histopathologic necrosis, % *	7.5 ± 9.7	18.6 ± 11.7	5.9 ± 8.1	<0.001	20.0 ± 10.3	5.3 ± 7.6	<0.001
Absence	54 (52.9)	2 (14.3)	51 (58.0)	0.002	1 (6.3)	52 (60.5)	<0.001
Presence	48 (47.1)	12 (85.7)	37 (42.0)		15 (93.7)	34 (39.5)	
Tumor differentiation				0.007			<0.001
Well	20 (19.6)	0 (0)	20 (22.7)		1 (6.3)	19 (22.1)	
Moderate	76 (74.5)	11 (78.6)	65 (73.9)		10 (62.4)	66 (76.7)	
Poor	6 (5.9)	3 (21.4)	3 (3.4)		5 (31.3)	1 (1.2)	
Lymphovascular invasion				0.368			0.348
Absence	36 (35.3)	3 (21.4)	33 (37.5)		4 (25.0)	32 (37.2)	
Presence	66 (64.7)	11 (78.6)	55 (62.5)		12 (75.0)	54 (62.8)	
Perineural invasion				0.122			>0.999
Absence	15 (14.7)	0 (0)	15 (17.0)		2 (12.5)	13 (15.1)	
Presence	87 (85.3)	14 (100)	73 (83.0)		14 (87.5)	73 (84.9)	
Tumor cellularity				0.019			0.017
< 50%	65 (63.7)	5 (35.7)	60 (68.2)		6 (37.5)	59 (68.6)	
≥ 50%	37 (36.3)	9 (64.3)	28 (31.8)		10 (62.5)	27 (31.4)	
Remaining acini				0.002			0.041
Absence	22 (21.6)	8 (57.1)	14 (15.9)		7 (43.8)	15 (17.4)	
Presence	80 (78.4)	6 (42.9)	74 (84.1)		9 (56.2)	71 (82.6)	
Lymph node metastasis				0.519			0.307
Absence	37 (36.3)	4 (28.6)	33 (37.5)		4 (25.0)	33 (38.4)	
Presence	65 (63.7)	10 (71.4)	55 (62.5)		12 (75.0)	53 (61.6)	

Values are presented as the number (%) of patients unless indicated otherwise. CT, computed tomography; MRI, magnetic resonance imaging; PDAC, pancreatic ductal adenocarcinoma. * Values are the mean ± standard deviation.

**Table 3:** Univariable and multivariable Cox proportional hazard analyses for disease-free survival after surgery.

Variable	Univariable Analysis		Multivariable Analysis	
	HR (95% CI)	*p* Value	HR (95% CI)	*p* Value
Age (≥65 years)	1.07 (0.70–1.64)	0.759		
Sex (male)	0.95 (0.62–1.46)	0.806		
CA 19-9 concentration (≥37 U/mL)	1.43 (0.87–2.33)	0.158		
Tumor size, cm	1.24 (1.06–1.44)	0.006	1.19 (1.01–1.41)	0.040
Tumor location				
Head/uncinate process	1 (reference)		1 (reference)	
Body or tail	0.49 (0.28–0.86)	0.013	0.46 (0.25–0.82)	0.009
T stage				
T1	1 (reference)			
T2 or T3	1.14 (0.69–1.89)	0.599		
Lymph node metastasis	1.62 (1.03–2.56)	0.038	1.27 (0.79–2.03)	0.325
Histopathologic necrosis, %	1.02 (0.99–1.04)	0.142		
Presence of imaging necrosis				
CT-detected necrosis	1.71 (0.93–3.16)	0.086		
MRI-detected necrosis	3.16 (1.77–5.65)	<0.001	2.64 (1.43–4.89)	0.002
Contact with SMV or PV	1.37 (0.89–2.12)	0.149		
Unenhanced T1WI hypointensity	1.07 (0.43–2.67)	0.877		
Pancreatic phase hypointensity	0.73 (0.35–1.53)	0.406		
Portal-venous phase hypointensity	1.02 (0.57–1.81)	0.955		
Delayed phase hypointensity	1.95 (1.18–3.20)	0.009	1.29 (0.75–2.23)	0.353
Diffusion restriction	2.20 (1.06–4.57)	0.035	2.10 (0.99–4.44)	0.053
Apparent diffusion coefficient (×10^−3^ mm^2^/s)	1.14 (0.72–1.81)	0.584		
Rim enhancement on MRI	1.03 (0.67–1.60)	0.885		

CA, carbohydrate antigen; CI, confidence interval; CT, computed tomography; HR, hazard ratio; MRI, magnetic resonance imaging; PV, portal vein; PDAC, pancreatic ductal adenocarcinoma; SMV, superior mesenteric vein; T1WI, T1-weighted image.

**Table 4:** Univariable and multivariable Cox proportional hazard analyses for overall survival after surgery.

Variable	Univariable Analysis		Multivariable Analysis	
	HR (95% CI)	*p* Value	HR (95% CI)	*p* Value
Age (≥ 65 years)	1.24 (0.74–2.07)	0.420		
Sex (male)	1.13 (0.68–1.88)	0.639		
CA 19-9 concentration (≥ 37 U/mL)	1.32 (0.74–2.35)	0.353		
Tumor size, cm	1.23 (1.02–1.48)	0.029	1.18 (0.97–1.44)	0.093
Tumor location				
Head/uncinate process	1 (reference)			
Body or tail	0.54 (0.28–1.04)	0.064		
T stage				
T1	1 (reference)			
T2 or T3	1.39 (0.76–2.53)	0.272		
Lymph node metastasis	1.75 (1.02–3.03)	0.044	1.64 (0.94–2.86)	0.084
Histopathologic necrosis, %	1.03 (1.00–1.05)	0.061		
Presence of imaging necrosis				
CT-detected necrosis	1.79 (0.90–3.56)	0.100		
MRI-detected necrosis	2.66 (1.40–5.06)	0.003	2.59 (1.35–4.97)	0.004
Contact with SMV or PV	1.61 (0.97–2.68)	0.067		
Unenhanced T1WI hypointensity	1.43 (0.315–5.92)	0.618		
Pancreatic phase hypointensity	1.29 (0.51–3.28)	0.587		
Portal-venous phase hypointensity	1.07 (0.55–2.06)	0.843		
Delayed phase hypointensity	1.77 (0.98–3.18)	0.058		
Diffusion restriction	1.94 (0.77–4.87)	0.157		
Apparent diffusion coefficient (× 10^−3^ mm^2^/s)	1.16 (0.68–1.99)	0.585		
Rim enhancement on MRI	0.89 (0.53–1.51)	0.676		

CA, carbohydrate antigen; CI, confidence interval; CT, computed tomography; HR, hazard ratio; MRI, magnetic resonance imaging; PV, portal vein; PDAC, pancreatic ductal adenocarcinoma; SMV, superior mesenteric vein; T1WI, T1-weighted image.

## Data Availability

All data accessed and analyzed in this study are available in the article and its Supplementary Materials.

## Supplementary Materials

The following supporting information can be downloaded at: https://www.mdpi.com/article/10.3390/cancers14225671/s1, Consistency of imaging necrosis between CT and MRI; Postoperative outcomes of PDAC with imaging necrosis according to CT contrast agent; Supplementary Table S1: MR sequence parameters; Supplementary Table S2: Histopathologic findings of PDAC according to CT-detected necrosis; Supplementary Table S3: Interreader agreement for imaging analysis; Supplementary Figure S1: A case of pancreatic ductal adenocarcinoma in a 71-year-old woman.

## References

[1] Siegel R.L., Miller K.D., Fuchs H.E., Jemal A. (2021). Cancer Statistics, 2021. CA Cancer J. Clin..

[2] Khorana A.A., Mangu P.B., Berlin J., Engebretson A., Hong T.S., Maitra A., Mohile S.G., Mumber M., Schulick R., Shapiro M. (2016). Potentially Curable Pancreatic Cancer: American Society of Clinical Oncology Clinical Practice Guideline. J. Clin. Oncol..

[3] Bengtsson A., Andersson R., Ansari D. (2020). The actual 5-year survivors of pancreatic ductal adenocarcinoma based on real-world data. Sci. Rep..

[4] Neoptolemos J.P., Stocken D.D., Dunn J.A., Almond J., Beger H.G., Pederzoli P., Bassi C., Dervenis C., Fernandez-Cruz L., Lacaine F. (2001). Influence of resection margins on survival for patients with pancreatic cancer treated by adjuvant chemoradiation and/or chemotherapy in the ESPAC-1 randomized controlled trial. Ann. Surg..

[5] Katz M.H., Wang H., Fleming J.B., Sun C.C., Hwang R.F., Wolff R.A., Varadhachary G., Abbruzzese J.L., Crane C.H., Krishnan S. (2009). Long-term survival after multidisciplinary management of resected pancreatic adenocarcinoma. Ann. Surg. Oncol..

[6] National Comprehensive Cancer Network Pancreatic Adenocarcinoma, Version 1. NCCN Clinical Practice Guidelines in Oncology Web Site.

[7] Khorana A.A., Mangu P.B., Berlin J., Engebretson A., Hong T.S., Maitra A., Mohile S.G., Mumber M., Schulick R., Shapiro M. (2017). Potentially Curable Pancreatic Cancer: American Society of Clinical Oncology Clinical Practice Guideline Update. J. Clin. Oncol..

[8] Nakatsura T., Hasebe T., Tsubono Y., Ryu M., Kinoshita T., Kawano N., Konishi M., Kosuge T., Kanai Y., Mukai K. (1997). Histological prognostic parameters for adenocarcinoma of the pancreatic head. Proposal for a scoring system for prediction of outcome. J. Hepato-Biliary Pancreat. Surg..

[9] Hiraoka N., Ino Y., Sekine S., Tsuda H., Shimada K., Kosuge T., Zavada J., Yoshida M., Yamada K., Koyama T. (2010). Tumour necrosis is a postoperative prognostic marker for pancreatic cancer patients with a high interobserver reproducibility in histological evaluation. Br. J. Cancer.

[10] Mitsunaga S., Hasebe T., Iwasaki M., Kinoshita T., Ochiai A., Shimizu N. (2005). Important prognostic histological parameters for patients with invasive ductal carcinoma of the pancreas. Cancer Sci..

[11] Kim D.W., Lee S.S., Kim S.O., Kim J.H., Kim H.J., Byun J.H., Yoo C., Kim K.P., Song K.B., Kim S.C. (2020). Estimating Recurrence after Upfront Surgery in Patients with Resectable Pancreatic Ductal Adenocarcinoma by Using Pancreatic CT: Development and Validation of a Risk Score. Radiology.

[12] Kim H., Kim D.H., Song I.H., Youn S.Y., Kim B., Oh S.N., Choi J.I., Rha S.E. (2022). Identification of intratumoral fluid–containing area by magnetic resonance imaging to predict prognosis in patients with pancreatic ductal adenocarcinoma after curative resection. Eur. Radiol..

[13] Hattori Y., Gabata T., Zen Y., Mochizuki K., Kitagawa H., Matsui O. (2010). Poorly enhanced areas of pancreatic adenocarcinomas on late-phase dynamic computed tomography: Comparison with pathological findings. Pancreas.

[14] Yoon S.E., Byun J.H., Kim K.A., Kim H.J., Lee S.S., Jang S.J., Jang Y.J., Lee M.G. (2010). Pancreatic ductal adenocarcinoma with intratumoral cystic lesions on MRI: Correlation with histopathological findings. Br. J. Radiol..

[15] Youn S.Y., Rha S.E., Jung E.S., Lee I.S. (2018). Pancreas ductal adenocarcinoma with cystic features on cross-sectional imaging: Radiologic-pathologic correlation. Diagn. Interv. Radiol..

[16] Kim J.H., Park S.H., Yu E.S., Kim M.H., Kim J., Byun J.H., Lee S.S., Hwang H.J., Hwang J.Y., Lee S.S. (2010). Visually isoattenuating pancreatic adenocarcinoma at dynamic-enhanced CT: Frequency, clinical and pathologic characteristics, and diagnosis at imaging examinations. Radiology.

[17] Lee S., Kim S.H., Park H.K., Jang K.T., Hwang J.A., Kim S. (2018). Pancreatic Ductal Adenocarcinoma: Rim Enhancement at MR Imaging Predicts Prognosis after Curative Resection. Radiology.

[18] Amin M.B., Greene F.L., Edge S.B., Compton C.C., Gershenwald J.E., Brookland R.K., Meyer L., Gress D.M., Byrd D.R., Winchester D.P. (2017). The Eighth Edition AJCC Cancer Staging Manual: Continuing to build a bridge from a population-based to a more “personalized” approach to cancer staging. CA Cancer J. Clin..

[19] Yoon S.H., Lee J.M., Cho J.Y., Lee K.B., Kim J.E., Moon S.K., Kim S.J., Baek J.H., Kim S.H., Kim S.H. (2011). Small (</= 20 mm) pancreatic adenocarcinomas: Analysis of enhancement patterns and secondary signs with multiphasic multidetector CT. Radiology.

[20] Punt C.J., Buyse M., Köhne C.H., Hohenberger P., Labianca R., Schmoll H.J., Påhlman L., Sobrero A., Douillard J.Y. (2007). Endpoints in adjuvant treatment trials: A systematic review of the literature in colon cancer and proposed definitions for future trials. J. Natl. Cancer Inst..

[21] Park H.S., Lee J.M., Choi H.K., Hong S.H., Han J.K., Choi B.I. (2009). Preoperative evaluation of pancreatic cancer: Comparison of gadolinium-enhanced dynamic MRI with MR cholangiopancreatography versus MDCT. J. Magn. Reson. Imaging.

[22] Lee J.K., Kim A.Y., Kim P.N., Lee M.G., Ha H.K. (2010). Prediction of vascular involvement and resectability by multidetector-row CT versus MR imaging with MR angiography in patients who underwent surgery for resection of pancreatic ductal adenocarcinoma. Eur. J. Radiol..

[23] Li D., Hu B., Zhou Y., Wan T., Si X. (2018). Impact of tumor size on survival of patients with resected pancreatic ductal adenocarcinoma: A systematic review and meta-analysis. BMC Cancer.

[24] Lau M.K., Davila J.A., Shaib Y.H. (2010). Incidence and survival of pancreatic head and body and tail cancers: A population-based study in the United States. Pancreas.

[25] Zheng Z., Wang M., Tan C., Chen Y., Ping J., Wang R., Liu X. (2019). Disparities in survival by stage after surgery between pancreatic head and body/tail in patients with nonmetastatic pancreatic cancer. PLoS ONE.

[26] Winer L.K., Dhar V.K., Wima K., Morris M.C., Lee T.C., Shah S.A., Ahmad S.A., Patel S.H. (2019). The Impact of Tumor Location on Resection and Survival for Pancreatic Ductal Adenocarcinoma. J. Surg. Res..

[27] Ghaneh P., Kleeff J., Halloran C.M., Raraty M., Jackson R., Melling J., Jones O., Palmer D.H., Cox T.F., Smith C.J. (2019). The Impact of Positive Resection Margins on Survival and Recurrence Following Resection and Adjuvant Chemotherapy for Pancreatic Ductal Adenocarcinoma. Ann. Surg..

[28] Konstantinidis I.T., Warshaw A.L., Allen J.N., Blaszkowsky L.S., Castillo C.F., Deshpande V., Hong T.S., Kwak E.L., Lauwers G.Y., Ryan D.P. (2013). Pancreatic ductal adenocarcinoma: Is there a survival difference for R1 resections versus locally advanced unresectable tumors? What is a “true” R0 resection?. Ann. Surg..

