# Supplementary material for: Survival Prediction after Curative Resection of Pancreatic Ductal Adenocarcinoma by Imaging-Based Intratumoral Necrosis
Source: Cancers (Basel). 2022 Nov 18;14(22):5671. doi: 10.3390/cancers14225671 (PMC9688323; doi:10.3390/cancers14225671)

Supplementary Material

Consistency of imaging necrosis between CT and MRI

For the detection of imaging necrosis, 90 (88.2%) PDAC patients were determined to be consistent between CT and MRI as follows: 9 patients showed imaging necrosis on both CT and MRI, whereas 81 patients showed no imaging necrosis. All 9 patients with necrosis on both CT and MRI had histopathological necrosis, and the mean diameter of the PDACs was 4.5 ± 0.7 cm.

Of the 12 (11.8%) PDAC patients with an inconsistency between CT and MRI, 7 patients showed MRI-detected necrosis but did not show CT-detected necrosis. Of the 7 patients, 6 had histopathological necrosis, and the mean diameter of the PDACs was 3.1 ± 1.0 cm. Among the remaining 5 patients who showed CT-detected necrosis but no MRI-detected necrosis, histopathological necrosis was found in 3 patients. The mean diameter of the PDACs was 4.0 ± 1.1 cm.

Postoperative outcomes of PDAC with imaging necrosis according to CT contrast agent

The DFS and OS rates tended to be poorer in patients with CT-detected necrosis using both CT contrast agents, although not statistically significant. In CT-Iobrix group, the mean DFS and OS of patients with CT-detected necrosis were 6.6 and 18.6 months, respectively, and those of patients without CT-detected necrosis were 14.6 and 34.2 months, respectively (DFS, $P = 0.105$; OS, $P = 0.082$). In CT-Ultravist group, the mean DFS and OS of patients with CT-detected necrosis were 16.9 and 37.1 months, respectively, and those of patients without CT-detected necrosis were 27.3 and 42.8 months, respectively (DFS, $P = 0.140$; OS, $P = 0.222$).

Supplementary Table S1. MR sequence parameters.

	Sequence				
	3D T1 GRE	Dual-echo T1 GRE	FSE T2	HASTE	DWI*
Repetition time (ms)	3.5	5.5	3488	500	7800
Echo time (ms)	1.2-1.4	1.3, OP; 2.6, IP	120	90	75
Flip angle (°)	15.5	9	140	150	90
Matrix	384×192	320×125	448×212	320×151	128×83
Field of view (mm)	380×297	380×297	380×300	380×299	380×309
Echo train length	1	1	29	256	1
Section thickness (mm)	3	3	6	6	6
No. of signal acquisitions	1	1	2	1	4

Parallel imaging was performed using a k-space-based technique (CAIPIRINHA, Siemens Healthineers; SENSE, Philips Healthcare).

MR, magnetic resonance; 3D, three-dimensional; T1, T1-weighted; GRE, gradient-recalled echo; FSE, fast spin-echo; T2, T2-weighted; HASTE, half-Fourier acquisition single-shot turbo spin-echo; DWI, diffusion-weighted imaging; OP, out-of-phase; IP, in-phase.

*Diffusion-weighted imaging was performed using a single-shot echo-planar imaging sequence with *b* values of 0 and 800 sec/mm^2.

Supplementary Table S2. Histopathologic findings of PDAC according to CT-detected necrosis.

Variable	Imaging necrosis on CT-Iobrix			Imaging necrosis on CT-Ultravist		
	Yes (n = 7)	No (n = 56)	*P*	Yes (n = 7)	No (n = 32)	*P*
Histopathologic necrosis (mean ± SD), %	20.0 ± 15.3	6.3 ± 8.2	<.001	17.1 ± 7.6	5.2 ± 8.0	.001
Absence	2 (28.6)	31 (55.4)		0 (0)	20 (62.5)	
Presence	5 (71.4)	25 (44.6)		7 (100)	12 (37.5)	
Tumor differentiation			.004			.445
Well	0 (0)	15 (26.8)		0 (0)	5 (15.6)	
Moderate	5 (71.4)	40 (71.4)		6 (85.7)	25 (78.1)	
Poor	2 (28.6)	1 (1.8)		1 (14.3)	2 (6.3)	
Lymphovascular invasion			.407			>.999
Absence	1 (14.3)	22 (39.3)		2 (28.6)	11 (34.4)	
Presence	6 (85.7)	34 (60.7)		5 (71.4)	21 (65.6)	
Perineural invasion			.337			>.999
Absence	0 (0)	11 (19.6)		0 (0)	4 (12.5)	
Presence	7 (100)	45 (80.4)		7 (100)	28 (87.5)	
Tumor cellularity			.001			>.999
< 50%	0 (0)	37 (66.1)		5 (71.4)	23 (71.9)	
≥ 50%	7 (100)	19 (33.9)		2 (28.6)	9 (28.1)	
Remaining acini			.007			.094
Absence	5 (71.4)	10 (17.9)		3 (42.9)	4 (12.5)	
Presence	2 (28.6)	46 (82.1)		4 (57.1)	28 (87.5)	

Lymph node metastasis			.699			.386
Absence	3 (42.9)	20 (35.7)		1 (14.3)	13 (40.6)	
Presence	4 (57.1)	36 (64.3)		6 (85.7)	19 (59.4)	

Values are presented as the number (%) of patients unless indicated otherwise.
PDAC, pancreatic ductal adenocarcinoma; CT, computed tomography; SD, standard deviation.

Supplementary Table S3. Interreader agreement for imaging analysis.

Imaging feature	Reviewer 1	Reviewer 2	Proportion of agreement (%)	Kappa value (95% confidence interval)
CT-detected necrosis	15	11	90/102 (88.2)	0.76 (0.64, 0.89)
MRI-detected necrosis	17	15	92/102 (90.2)	0.80 (0.69, 0.92)
Contact to SMV or PV	43	37	76/102 (74.5)	0.49 (0.32, 0.66)
Unenhanced T1WI hypointensity	97	93	90/102 (88.2)	0.76 (0.64, 0.89)
Pancreatic phase hypointensity	94	92	88/102 (86.3)	0.73 (0.59, 0.86)
Portal venous phase hypointensity	84	86	84/102 (82.4)	0.65 (0.50, 0.80)
Delayed phase hypointensity	70	69	83/102 (81.4)	0.63 (0.48, 0.78)
Diffusion restriction	89	80	87/100* (87.0)	0.74 (0.61, 0.87)
ADC ($\times 10^{-3}$ mm^2/sec)	1.37 ± 0.38†	1.36 ± 0.41†	NA	0.89 (0.83, 0.92)‡
Rim enhancement on MRI	50	20	70/102 (68.6)	0.37 (0.19, 0.55)

Data represent the number of patients with imaging features.

CT, computed tomography; MRI, magnetic resonance imaging; SMV, superior mesenteric vein; PV, portal vein; T1WI, T1-weighted imaging; ADC, apparent diffusion coefficient; NA, not applicable; PDAC, pancreatic ductal adenocarcinoma.

*Diffusion-weighted imaging was unavailable for two patients.

†Data are expressed as the mean ± standard deviation.

‡Agreement was evaluated using the intraclass correlation coefficient, with its 95% confidence interval in parentheses.

Supplementary Figure S1. Pancreatic ductal adenocarcinoma in the head of the pancreas in a 71-year-old woman. (a) An axial contrast-enhanced CT image shows a 4.3-cm hypoenhancing pancreatic head mass (arrowheads) without CT detection of necrosis. (b) An axial T2-weighted MR image with fat suppression shows a pancreatic head mass (arrowheads) accompanied by an intratumoral fluid-containing area at the central location (arrow). On axial dynamic contrast-enhanced T1-weighted MR images, (c) portal-venous and (d) delayed phases show a hypoenhancing pancreatic head mass (arrowheads) with nonenhanced intratumoral tissue judged as MRI-detected necrosis (arrow). (e, f) Micrograph shows gland-forming carcinoma cells in the periphery (black arrowheads) and necrosis (asterisks) in the central area of the tumor (hematoxylin and eosin stain, ×12.5 (e) and ×200 (f)). In this patient, imaging necrosis on CT and MRI was judged to be inconsistent, but histopathologically, approximately 30% of tumors consisted of necrosis. Distant lymph node metastasis was found on CT 3 months after margin-negative resection (not shown), and the patient died 2 years later.

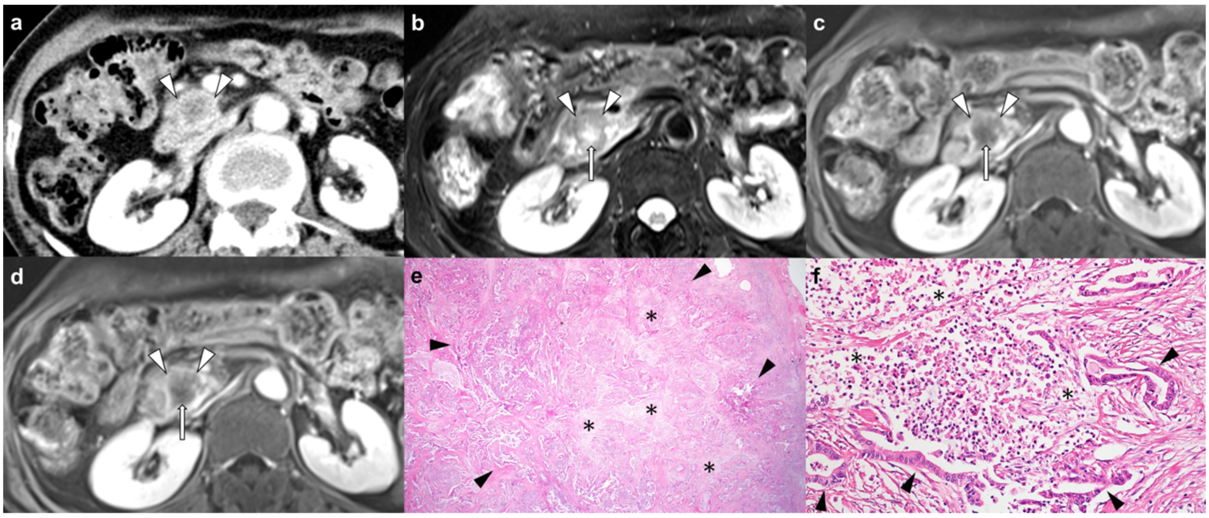

Supplement: Supplementary file 1 [file cancers-14-05671-s001.zip › cancers-2015793-supplementary.pdf]
